# 

**Published:** 1874-06

**Authors:** 


### CONTENTS:

ORIGINAL COMMUNICATIONS:
ARTICLE I.—Two Cases in which the Pneumatic Aspirator was employed successfully. By E. W. Lee, M.D... 321
ART. II.—Case of Umbilical Hernia, with Loss of Portion of Intestine. By J. N. Reeder, M.D.. ... 323
ART. III.—Case of Habitual Regurgitation, Simulating Rumination in the Lower Animals. By D. W. Graham, M. D... 326
ART. IV.—A Complete Closure of Hymen. By G. C. Paoli, M.D... 328

PROGRESS IN MEDICAL SCIENCES:
ART. I.—Report on Pathology and Microscopy. By I. N. Danforth, M.D.. 328
ART. II.—Progress of Obstetrics and Gynæcology. By A. Reeves Jackson, M.D... 333
ART. III.—Progress in Therapeutics. By J. H. Etheridge, M.D... 337
REPORTS OF SOCIETIES... 348
CLINICS... 369
EDITORS' BOOK TABLE... 371
EDITORIAL... 377

